# Multiple Stressors in Agricultural Streams: A Mesocosm Study of Interactions among Raised Water Temperature, Sediment Addition and Nutrient Enrichment

**DOI:** 10.1371/journal.pone.0049873

**Published:** 2012-11-21

**Authors:** Jeremy J. Piggott, Katharina Lange, Colin R. Townsend, Christoph D. Matthaei

**Affiliations:** Department of Zoology, University of Otago, Dunedin, New Zealand; University of Southampton, United Kingdom

## Abstract

Changes to land use affect streams through nutrient enrichment, increased inputs of sediment and, where riparian vegetation has been removed, raised water temperature. We manipulated all three stressors in experimental streamside channels for 30 days and determined the individual and pair-wise combined effects on benthic invertebrate and algal communities and on leaf decay, a measure of ecosystem functioning. We added nutrients (phosphorus+nitrogen; high, intermediate, natural) and/or sediment (grain size 0.2 mm; high, intermediate, natural) to 18 channels supplied with water from a nearby stream. Temperature was increased by 1.4°C in half the channels, simulating the loss of upstream and adjacent riparian shade. Sediment affected 93% of all biological response variables (either as an individual effect or via an interaction with another stressor) generally in a negative manner, while nutrient enrichment affected 59% (mostly positive) and raised temperature 59% (mostly positive). More of the algal components of the community responded to stressors acting individually than did invertebrate components, whereas pair-wise stressor interactions were more common in the invertebrate community. Stressors interacted often and in a complex manner, with interactions between sediment and temperature most common. Thus, the negative impact of high sediment on taxon richness of both algae and invertebrates was stronger at raised temperature, further reducing biodiversity. In addition, the decay rate of leaf material (strength loss) accelerated with nutrient enrichment at ambient but not at raised temperature. A key implication of our findings for resource managers is that the removal of riparian shading from streams already subjected to high sediment inputs, or land-use changes that increase erosion or nutrient runoff in a landscape without riparian buffers, may have unexpected effects on stream health. We highlight the likely importance of intact or restored buffer strips, both in reducing sediment input and in maintaining cooler water temperatures.

## Introduction

Understanding the interactive and cumulative effects of multiple anthropogenic stressors is a pressing challenge [1], [2] because ‘ecological surprises’ may result if stressors interact in unexpected ways [3]. Stressors can act in a predictable, ‘additive’ manner or yield complex synergistic or antagonistic responses [4], and researchers have turned to empirical surveys, field experiments and laboratory experiments to unravel their individual and combined effects [5].

Agriculture affects streams worldwide, particularly through nutrient enrichment and increased fine sediment input [6]. In New Zealand, this is associated with land conversion to pasture or agricultural intensification to deer and dairy farming [7]. Increased water temperature can also be expected to act as a stressor in many freshwater communities. Its effect is likely to increase due to agricultural intensification that reduces riparian shade [8] as well as anthropogenic climate change [9]. Thus, it will be important to understand whether and how rising temperature interacts with other stressors.

Our objective was to determine the individual and combined effects of nutrient enrichment, increased fine sediment on the bed and raised water temperature on stream community structure and function. We focused on the responses of three key components of stream ecosystems (aquatic invertebrates, benthic algae and organic matter decomposition) in experimental streamside channels using a factorial design. We chose stressor levels simulating those found in Southern New Zealand pasture, deer and dairy farming streams [10] in situations with or without tussock riparian shading. To our knowledge, this is the first time these three stressors, which can be expected to affect streams worldwide, have been manipulated simultaneously.

While individual stressor effects may follow a linear negative response gradient, others may follow non-linear threshold [11] or subsidy-stress [12] patterns, where lower stressor levels have neutral or positive effects, respectively, but with the effect becoming negative beyond a threshold [13]. Based on our previous studies and others [14]–[16], we predicted that the individual effects of nutrient enrichment and raised water temperature on biological response variables would generally be positive (increased invertebrate density, algal biomass/cell density and organic matter decay rates), but with changes to community composition because taxa that tolerate elevated nutrient levels or temperatures will be favoured. We further predicted sediment addition to be a particularly pervasive stressor [5]. In contrast to most published literature, we expected higher organic decay rates under sediment, as detected in an earlier experiment [17]. The interactive effects among raised temperature and the other stressors are largely unknown [9].

## Results

### Invertebrates

**Figure 1:**
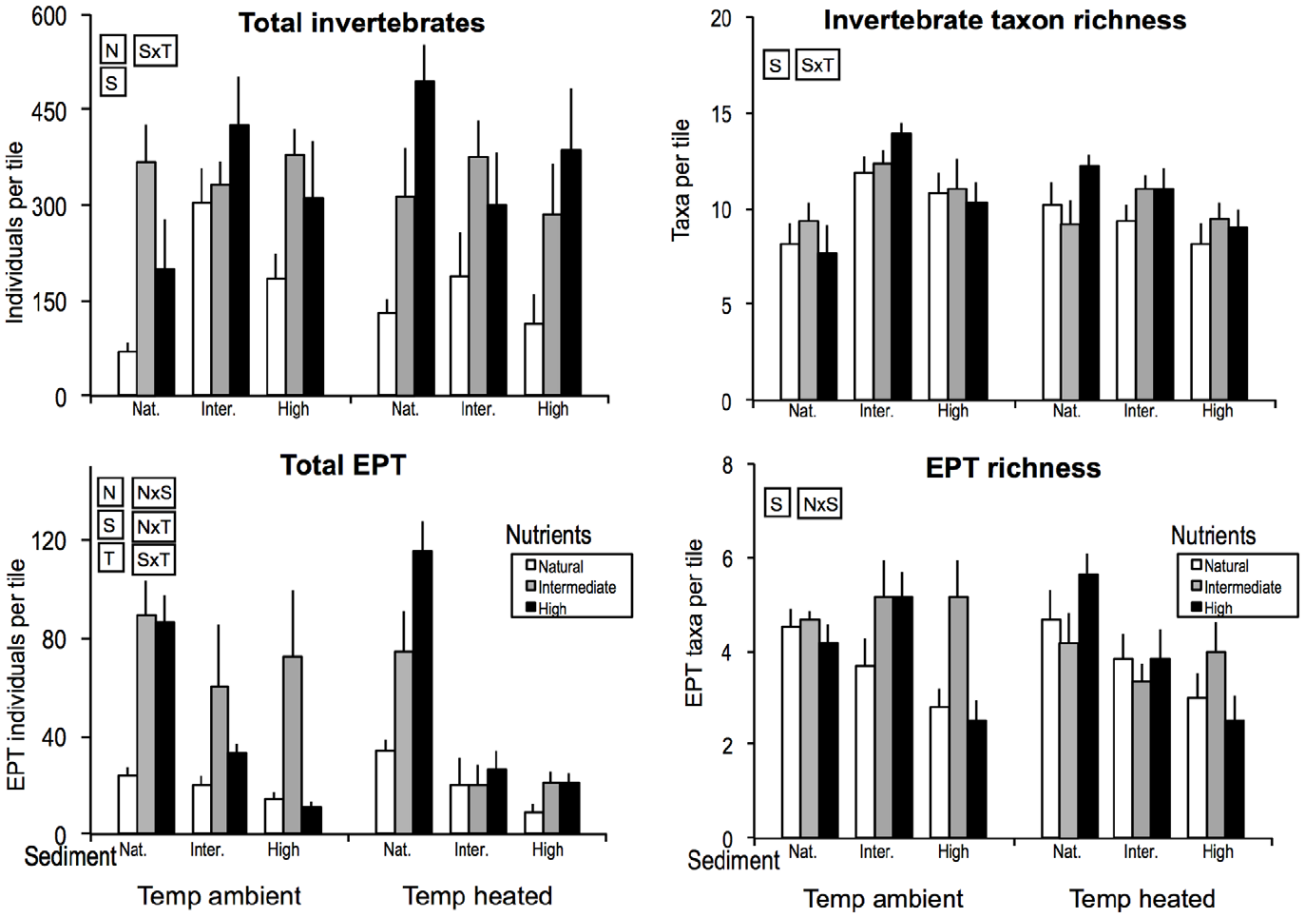
Averages of invertebrate community variables across the experimental treatments sampled on days 18 and 30 combined. Error bars (SEs) show the within-channel variation (n = 3) that is represented by the factor ‘sample’ in the statistical analysis (see text). Text in rectangles indicates significant single-factor effects (nutrients: N, sediment: S and temperature: T) or interactions.

Augmentation of nutrients and of sediment at intermediate and high levels increased total invertebrate abundance overall (i.e. based on the main effects of these factors in the analysis; Table 1). Added sediment also affected total abundance via an interaction with temperature (Fig. 1), having a positive effect at ambient temperature but a negative effect at raised temperature. Total abundance of EPT (Ephemeroptera, Plecoptera and Trichoptera; i.e., nymphs of mayflies, caddisflies and stoneflies) was higher in channels with nutrient enrichment but lower in channels with sediment addition (Table 1, Fig. 1). Nutrients affected EPT abundance via two-way interactions with both sediment (strongest increase with rising nutrients in channels without added sediment) and temperature (peaking at intermediate nutrients at ambient temperature but not at raised temperature). Finally, sediment interacted with temperature, with a stronger negative effect of added sediment at raised temperature.

**Table 1:** Invertebrate responses to the experimental treatments.

Dependent variable	%	Nutrients	Ranking	Sediment	Ranking	Temperature	Ranking	Nutrients × sediment	Nutrients × temperature	Sediment × temperature
Total invertebrates		**<0.001** (0.48)	N<(I = H)	**<0.001** (0.49)	N<(I = H)	0.44		0.49	0.86	**0.004** (0.34)
Total EPT		**<0.001** (0.65)	N<(I = H)	**<0.001** (0.80)	N>(I = H)	**0.01** (0.19)	SI	**0.01** (0.36)	**0.003** (0.35)	**0.02** (0.26)
Invert’ taxon richness		0.22		**0.002** (0.36)	SI	0.27		0.79	0.62	**0.002** (0.37)
EPT richness		0.14		**<0.001** (0.40)	(N = I)>H	0.17		**0.01** (0.38)	0.07	0.08
Community composition (MANOVA; 11 taxa)	98.0	**0.04** (0.54)		**<0.001** (0.85)		0.47		0.14	0.11	**0.04** (0.54)
Chironomidae	54.2	**<0.001** (0.51)	N<(I = H)	0.14		0.71		0.52	**0.02** (0.25)	**0.009** (0.29)
Cladocera	6.7	0.26		**<0.001** (0.54)	N<I	0.50		0.42	0.11	0.08
Copepoda	4.9	0.12		**0.001** (0.39)	I>H	0.48		0.39	0.22	**0.03** (0.23)
*Deleatidium* spp.	2.0	0.07		**<0.001** (0.56)	N>(I = H)	**0.01** (0.21)	Amb>Heat	0.14	0.06	0.80
*Hydora* spp.	1.2	0.47		**0.006** (0.31)	N<I	0.59		0.79	0.72	0.48
Hydrobiosidae	0.5	**0.04** (0.21)	SI	0.75		0.05 (0.14)		0.17	**0.003** (0.34)	0.23
Nematoda	4.7	0.35		**<0.001** (0.71)	N<(I = H)	**0.02** (0.18)	Amb<Heat	0.75	0.05 (0.20)	0.20
*Oxyethira* spp.	6.9	**0.02** (0.26)	SI	0.05 (0.19)		0.27		0.49	**0.01** (0.29)	**0.001** (0.40)
*P. antipodarum*	10.1	**0.03** (0.22)	N<I	**<0.001** (0.57)	(N = H)<I	0.86		0.92	0.15	0.17
Conoescidae	5.2	**<0.001** (0.53)	N<(I = H)	**<0.001** (0.80)	N>(I = H)	0.27		**0.001** (0.49)	**0.02** (0.27)	0.47
Tanypodinae	1.6	0.66		0.08		0.37		0.88	0.89	**0.02** (0.25)

Summary (*P*-values) of repeated-measures (M)ANOVAs comparing between-subjects invertebrate responses between experimental treatments. MANOVA *P*-values are for the Pillai’s Trace statistic. Rankings for *post hoc* tests in cases with significant effects are given in columns 4, 6 and 8, and relative abundances of taxa in column 2. Nutrient and sediment treatments: N, natural; I, intermediate; H, high. Temperature: Amb, ambient; Heat, heated. *P*-values <0.05 are in bold print. Effect sizes are shown in parentheses for cases where *P*≤0.05. ‘SI’ indicates cases where a substantial interaction between two factors (effect size of interaction term larger than the size of the corresponding main effects) prevented an overall post-doc ranking for the main effect in question.

At ambient temperature, total invertebrate taxon richness showed a subsidy-stress response to sediment addition, with highest richness at intermediate sediment levels (Table 1, Fig. 1). At raised temperature, however, total richness declined consistently with sediment, resulting in a marked sediment by temperature interaction. EPT taxon richness decreased at high sediment levels overall but not at intermediate nutrient concentrations, as indicated by a negative main effect of sediment and a nutrient by sediment interaction (Table 1, Fig. 1).

**Figure 2:**
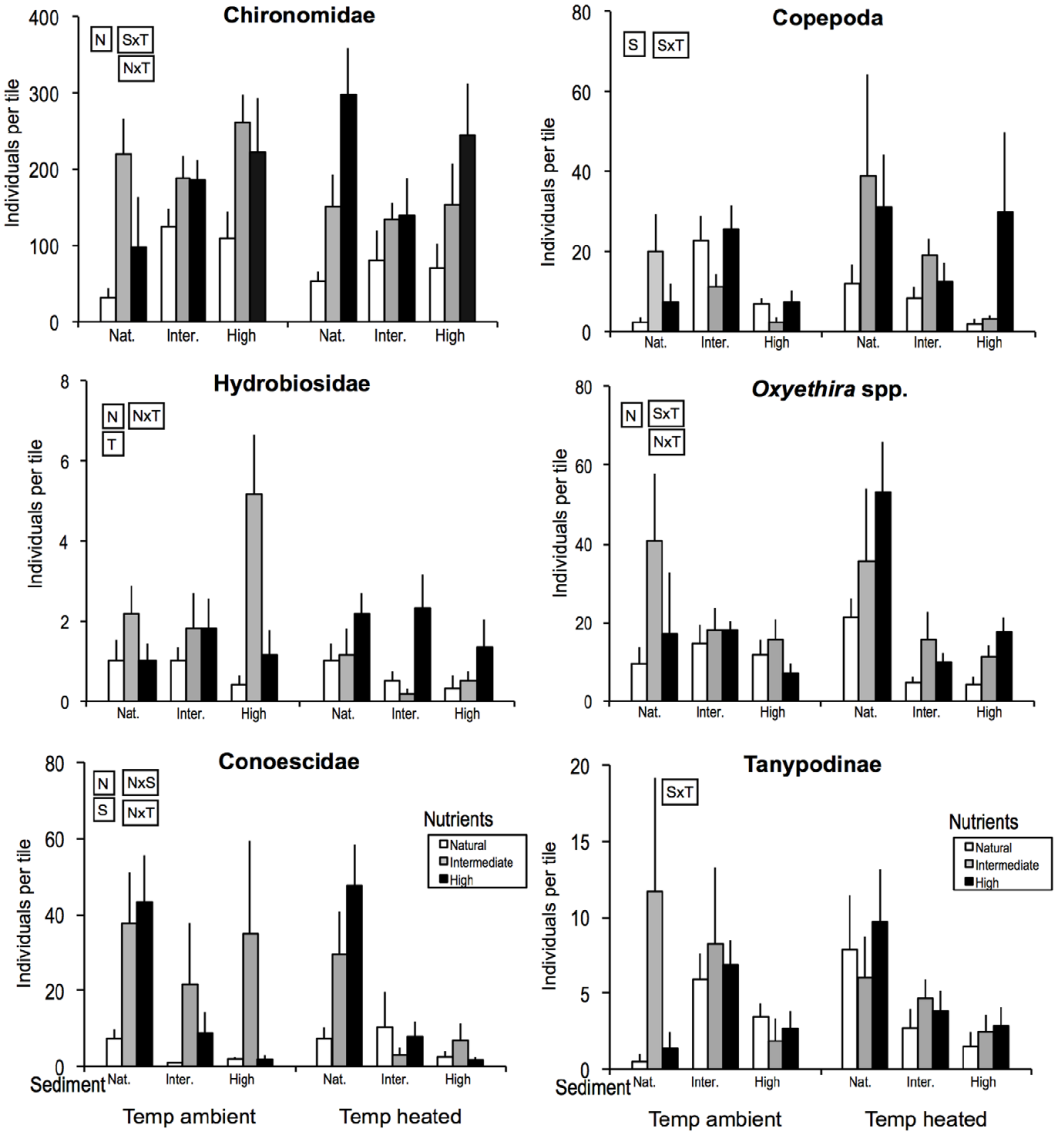
Abundance patterns (averages +SEs) for common invertebrate taxa (days 18 and 30 combined) that showed significant interactions between paired manipulated factors. For more details see Fig. 1.

The invertebrate MANOVA revealed that invertebrate community composition was unaffected by raised water temperature but differed across nutrient and sediment treatments overall, and that sediment and temperature interacted (Table 1). Focusing on the commonest 11 taxa (comprising 98% of the community; Table 1), raised water temperature increased the abundance of nematode worms but decreased nymphs of the mayfly *Deleatidium*. Nutrient enrichment at intermediate or high levels resulted in higher abundances of chironomid midge larvae (excluding Tanypodinae), the snail *Potamopyrgus antipodarum* (Gray) and Conoescidae caddis larvae. Sediment addition at intermediate or high levels decreased abundances of *Deleatidium* and Conoescidae (and Copepoda were less common at high than intermediate sediment) but increased abundances of Cladocera, the beetle *Hydora*, Nematoda and Tanypodinae, while *P. antipodarum* peaked at intermediate sediment. Temperature and nutrient effects interacted for Hydrobiosidae, Conoescidae, the caddis *Oxyethira* and Chironomidae (Fig. 2), with abundances peaking in channels with intermediate nutrient enrichment at ambient temperature but in highly enriched channels at raised temperature. Sediment and temperature effects also interacted for four taxa: Chironomidae became more common with rising sediment levels at ambient but not at raised temperature while Copepoda, *Oxyethira* and Tanypodinae all became consistently rarer with increasing sediment at raised temperature but not at ambient temperature (where neutral or subsidy-stress patterns for sediment occurred). Finally, nutrients and sediment interacted for Conoescidae, whose abundance generally decreased with sediment addition and increased with nutrient enrichment, except at high sediment where high nutrient levels had a neutral effect.

### Leaf Decomposition

**Figure 3:**
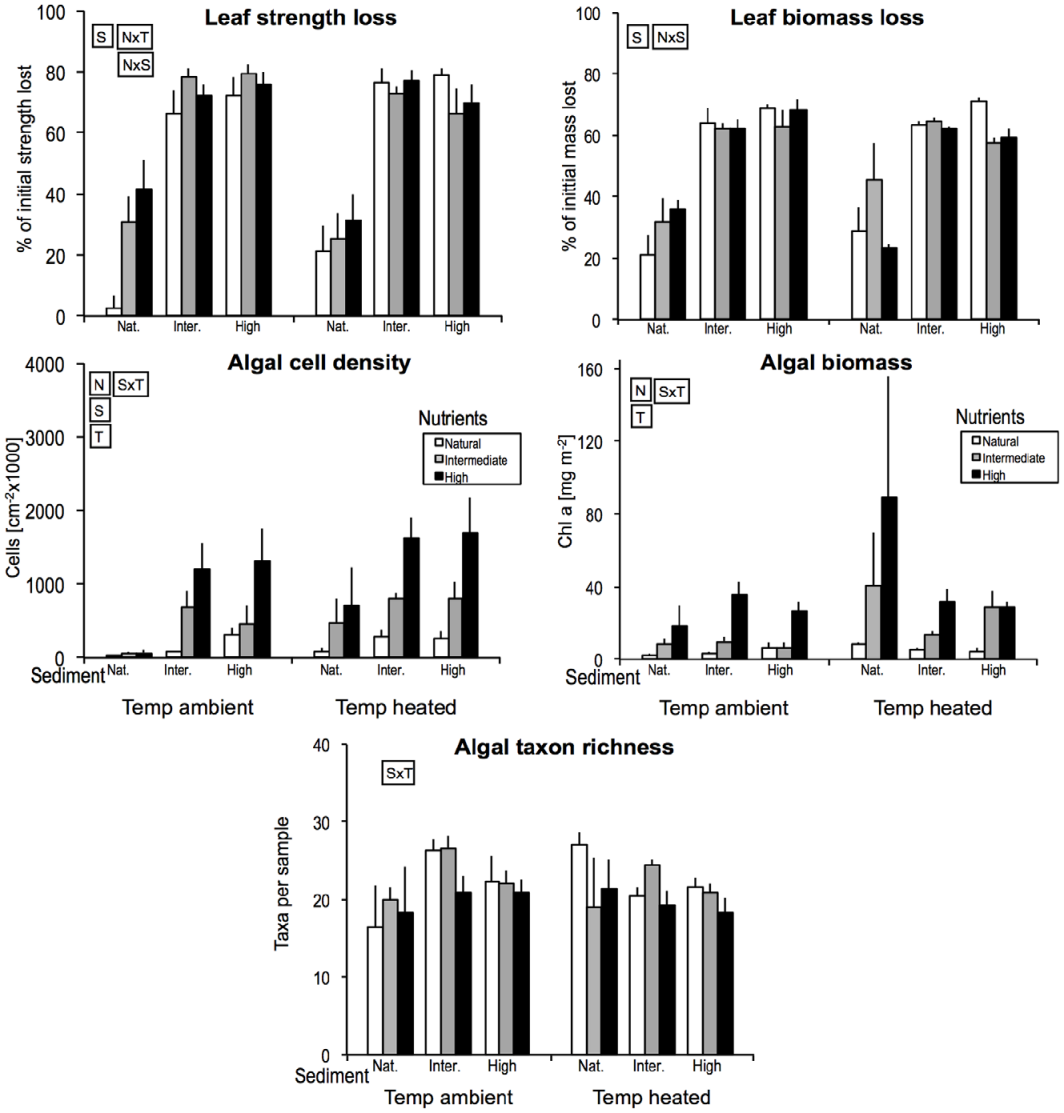
Averages of leaf decay variables (sampled on day 18) and algal community/biomass variables (sampled on day 29) across the experimental treatments. For more details see Fig. 1.

Both leaf mass loss and strength loss were greater at intermediate and high sediment levels than in channels without sediment addition but, as main effects, neither temperature nor nutrients affected leaf decomposition significantly (Table 2, Fig. 3). Temperature and nutrient effects on strength loss interacted, with a positive effect of nutrient enrichment at ambient but not at raised temperature. Sediment and nutrient effects on strength loss also interacted, with a positive effect of nutrient enrichment occurring mainly in channels without added sediment. Leaf mass loss showed no interactions between paired stressors.

**Table 2:** Leaf decomposition and algal responses to the experimental treatments.

Dependent variable	%	Nutrients	Ranking	Sediment	Ranking	Temperature	Ranking	Nutrients × sediment	Nutrients × temperature	Sediment × temperature
Leaf strength loss		0.05 (0.19)		**<0.001** (0.90)	N<(I = H)	0.998		**0.03** (0.30)	**0.02** (0.24)	0.57
Leaf mass loss		0.75		**<0.001** (0.85)	N<(I = H)	0.98		0.06	0.15	0.53
Algal cell density		**<0.001** (0.57)	N<(I = H)	**<0.001** (0.70)	N<(I = H)	**<0.001** (0.34)	Amb<Heat	0.72	0.77	**0.02** (0.24)
Algal biomass		**<0.001** (0.78)	N<I<H	0.95		**<0.001** (0.37)	Amb<Heat	0.94	0.27	**0.03** (0.21)
Algal taxon richness		0.18		0.18		0.88		0.58	0.62	**0.04** (0.21)
Community composition (MANOVA; 13 taxa)	82.7	**0.003** (0.68)		**0.03** (0.61)		**0.04** (0.67)		**0.04** (0.52)	0.14	0.42
*Nitzschia amphibia*	1.4	**0.04** (0.21)	N<H	**<0.001** (0.42)	N<(I = H)	**0.02** (0.18)	Amb<Heat	0.48	0.63	0.82
*Navicula cryptocephala*	1.6	0.07		**<0.001** (0.50)	N<(I = H)	0.12		**0.01** (0.36)	0.93	0.11
*Fragilaria vaucheriae*	1.8	0.73		**0.04** (0.21)	N<I	0.25		0.33	0.27	0.38
*Gomphonema minutum*	2.2	0.35		**<0.001** (0.40)	N>(I = H)	0.29		0.46	0.51	**0.02** (0.24)
*Nitzschia dissipata*	6.8	**<0.001** (0.44)	N<(I = H)	**<0.001** (0.65)	N<(I = H)	0.72		0.65	0.37	0.70
*Gloeocystis* sp.	3.0	0.59		**0.03** (0.22)	N<I	0.20		0.49	0.59	0.52
*Cylindrospermum* sp.	7.4	0.63		**<0.001** (0.56)	N<(I = H)	**0.003** (0.28)	Amb<Heat	0.12	0.56	0.40
*Navicula capitoradiata*	4.5	**0.045** (0.20)	N<(I = H)	**<0.001** (0.67)	N<(I = H)	0.22		0.49	0.73	0.28
*Ankistrodesmus* spp.	1.4	**0.02** (0.24)	SI	**0.002** (0.36)	N<(I = H)	0.97		0.39	**0.003** (0.34)	0.18
*Scenedesmus* spp.	25.74	**0.002** (0.36)	N<(I = H)	**<0.001** (0.49)	N<(I = H)	0.57		0.40	0.17	0.23
*Nitzschia palea*	16.5	**0.004** (0.32)	N<(I = H)	**<0.001** (0.72)	N<(I = H)	**0.009** (0.22)	Amb<Heat	0.89	0.07	0.07
*Navicula cryptotenella*	7.0	0.06		**<0.001** (0.66)	N<(I = H)	0.06		0.39	0.70	0.16
*Encyonema minuta*	3.4	**0.01** (0.31)	N<(I = H)	**<0.001** (0.55)	N<(I = H)	**0.009** (0.22)	SI	0.78	0.28	**0.007** (0.30)
Algal growth forms (MANOVA; 5 groups)	100	**<0.001** (0.41)		**<0.001** (0.48)		0.69		**0.03** (0.21)	0.38	0.30
Filamentous	21.0	**<0.001** (0.39)	N>(I = H)	**0.02** (0.24)	N>I	0.67		0.10	0.08	**0.049** (0.19)
Erect or stalked	6.7	0.31		0.80		0.45		0.12	0.45	0.34
Adnate or prostrate	20.4	0.64		**<0.001** (0.71)	N>(I = H)	0.64		0.94	0.35	0.66
Metaphyton	18.1	**<0.001** (0.58)	N<I<H	**<0.001** (0.43)	SI	0.27		**<0.001** (0.55)	0.88	0.32
Motile	33.9	0.86		**<0.001** (0.80)	N<(I = H)	0.74		0.42	0.47	0.42

Summary (*P*-values) of nested (M)ANOVAs comparing between-subjects leaf decomposition and algal response variables between nutrient, sediment and temperature treatments. See Table 1 for further details.

### Algae

Overall, algal cell density increased with nutrient or sediment levels and raised temperature, and algal biomass accrual (chlorophyll a) showed the same patterns for nutrients and temperature but was unaffected by added sediment (Table 2, Fig. 3). Temperature and sediment effects interacted for both algal variables, with the positive effect of added sediment being stronger at ambient than at raised temperature for cell density and reversed at raised temperature for biomass. Algal taxon richness was affected only by an interaction of temperature and sediment. At ambient temperature, richness demonstrated a subsidy-stress response to sediment with richness peaking when sediment was intermediate. By contrast, richness declined consistently with sediment at raised temperature (Fig. 3).

The algal taxa MANOVA showed that community composition changed overall with nutrient enrichment, sediment addition and raised temperature, and that nutrient and sediment effects interacted (Table 2). Focusing on the 13 commonest taxa, raised water temperature increased overall densities of *Nitzschia amphibia, Cylindrospermum* and *Nitzschia palea*. Further, nutrient enrichment to any level caused increased densities of *N. amphibia, Nitzschia dissipata, Navicula capitoradiata, Scenedesmus, N. palea* and *E. minuta* (Table 2). Sediment affected all the common taxa, with 12 being more abundant in channels with intermediate and/or high addition but *Gomphonema minutum* showing the opposite pattern.

**Figure 4:**
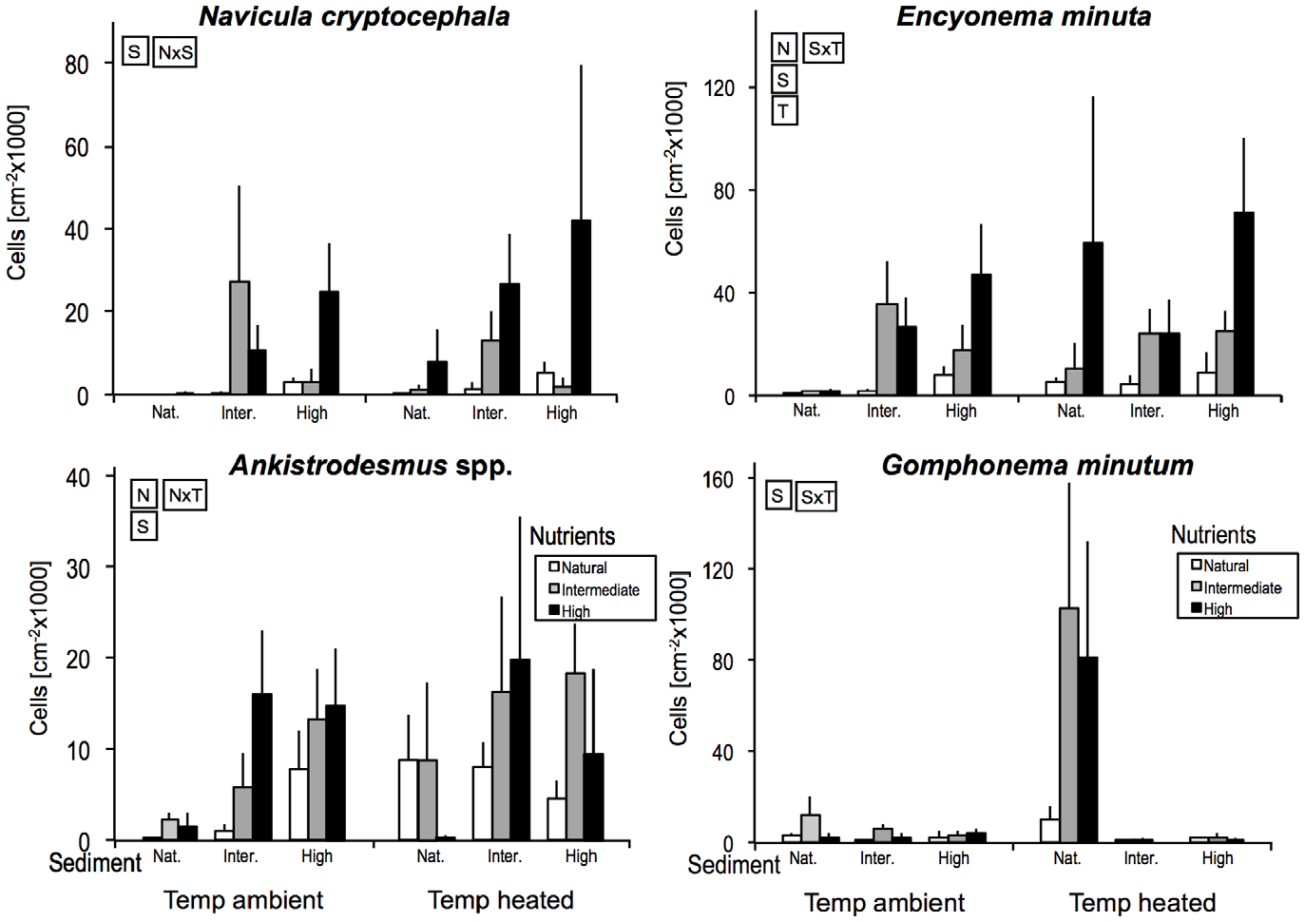
Density patterns (averages +SEs) for the common benthic algal taxa sampled on day 29 that showed significant interactions between paired manipulated factors. For more details see Fig. 1.

Temperature and nutrients had interactive effects on *Ankistrodesmus*, with densities increasing consistently with rising nutrient levels at ambient temperature, whereas densities peaked at intermediate nutrient concentrations when temperature was raised (Fig. 4). Temperature and sediment interacted for *G. minutum* and *E. minuta*, but the two taxa were affected quite differently (Fig. 4). For the former, the negative effect of added sediment was stronger at raised than at ambient water temperature. By contrast, the positive effect of sediment on the latter taxon was weaker at raised water temperature. Finally, an interaction between nutrients and sediment influenced *Navicula cryptocephala*, with high nutrient enrichment increasing densities markedly at the top sediment level but only weakly at the two lower sediment levels (Fig. 4).

The algal growth form MANOVA showed overall effects of nutrients and sediment on algal functional organisation, but no effect of temperature (Table 2). The between-subjects effects revealed that nutrient enrichment at intermediate or high levels decreased the overall prevalence of filamentous algae, whereas it increased representation of metaphytic algae. Sediment addition at intermediate or high levels decreased the relative abundance of adnate/prostrate algae while the representation of metaphytic and motile algae increased. Further, intermediate (but not high) sediment addition decreased filamentous algae.

**Figure 5:**
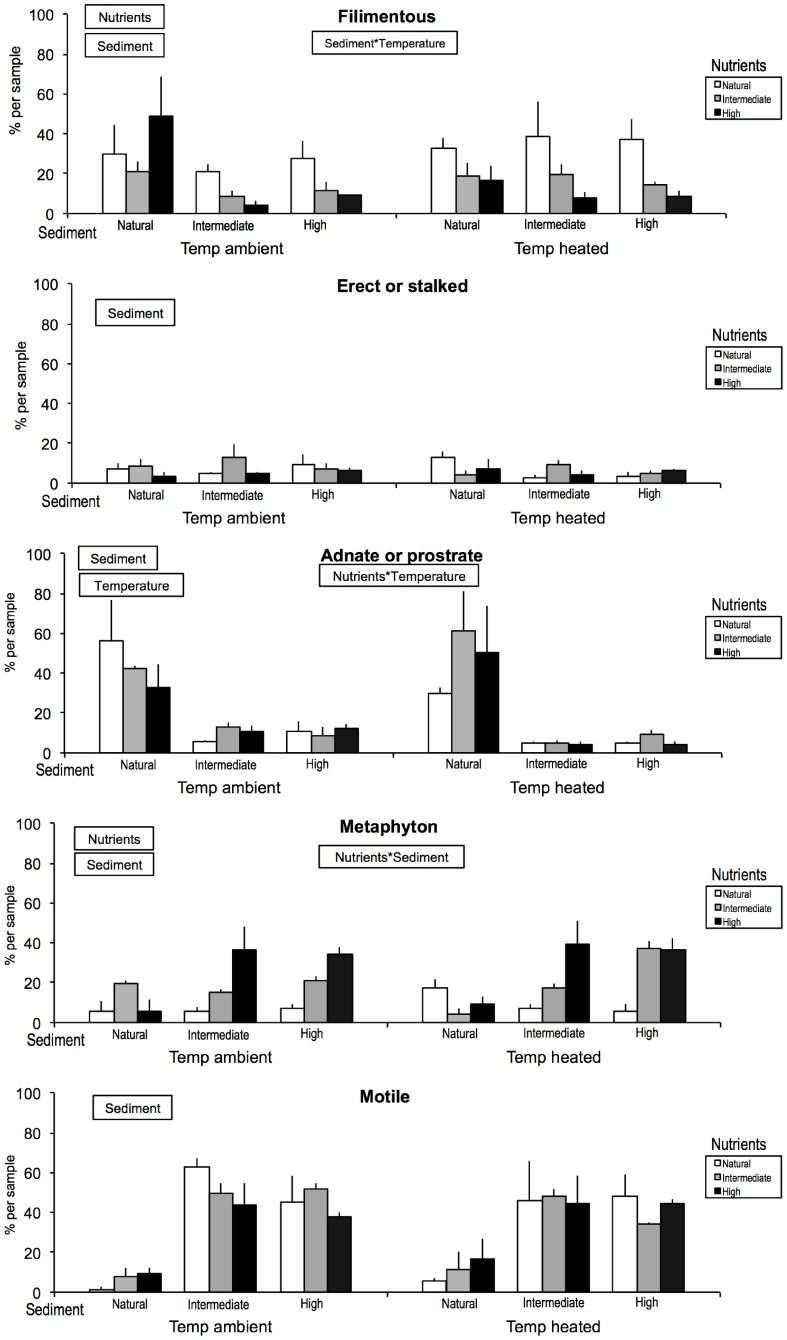
Relative abundance patterns (averages +SEs) for the benthic algal growth form classifications sampled on day 29. For more details see Fig. 1.

Temperature and sediment effects interacted for filamentous algae, with a negative effect of added sediment at ambient temperature but not at raised temperature (Fig. 5). Finally, nutrients and sediment interacted for metaphytic algae, with the positive effect of nutrient enrichment being evident only in channels with added sediment (Fig. 5).

## Discussion

### The Three Stressors Compared

Of the three land-use related stressors that we studied, sediment affected 78% of all 41 biological response variables (as an interpretable main effect), nutrient enrichment affected 44% and temperature affected 20% (Table 3). Not only were sediment effects most prevalent, according to average effect sizes they were also the most intense (Table 3). These results add weight to previous conclusions about the influence of nutrient enrichment and, in particular, the widespread and powerful effects of sediment addition on stream communities [5], [13], [17]. The most novel aspect of the current study has been to incorporate a small rise in stream temperature to simulate the influence of removal of riparian vegetation. This third stressor also influenced a variety of biological variables as a main effect and, even more importantly, had interactive effects in combination with the other stressors (discussed in the next section).

**Table 3:** Overview of results for biological response variables.

	Nutrients	Sediment	Temperature	Nutrients × sediment	Nutrients × temperature	Sediment × temperature
Invertebrates	6	11	2	3	5	8
(16 variables)	37.5%	68.8%	12.5%	18.8%	31.3%	50.0%
Mean effect size	0.49±0.06	0.58±0.06	0.20±0.02	0.41±0.04	0.33±0.04	0.32±0.04
Leaf decay	0	2	0	1	1	0
(2 variables)		100.0%		50.0%	50.0%	
Mean effect size		0.88±0.03		0.3	0.24	
Algae	12	19	6	4	1	6
(23 variables)	52.2%	82.6%	26.1%	17.4%	4.3%	26.1%
Mean effect size	0.36±0.06	0.50±0.05	0.19±0.06	0.41±0.08	0.44±0.09	0.22±0.02
**OVERALL**	**18**	**32**	**8**	**8**	**7**	**14**
(41 variables)	**43.9%**	**78.0%**	**19.5%**	**19.5%**	**17.1%**	**34.1%**
**Mean effect size**	**0.39±0.05**	**0.55±0.04**	**0.19±0.05**	**0.40±0.04**	**0.34±0.03**	**0.27±0.02**

Numbers and percentages of interpretable significant main effects (i.e. not including cases where a factor is involved in a substantial interaction with another; see Table 1) and significant interactions for the three categories of biological response variable. Means of effect sizes ± standard errors are shown.

As predicted, added fine sediment was the most pervasive stressor, negatively affecting abundances of sensitive EPT taxa (e.g. *Deleatidium* and Conoescidae), but increasing the abundance of total invertebrates and nematode worms, a taxon known to favour sediment-rich habitats [18]. Invertebrate taxon richness and several other invertebrate variables responded positively to sediment addition up to intermediate levels, probably reflecting the increased habitat heterogeneity provided by a moderate amount of fine sediment. However, at high sediment levels, negative effects tended to predominate, in accordance with findings of Matthaei et al. [17]. Many invertebrates are sensitive to clogging of respiratory apparatus, reductions in feeding efficiency and loss of appropriate habitat when sediment levels are high [19]. The diatom *Gomphonema minutum*, a genus known to strongly prefer smooth substrata [20], was the only common alga to respond negatively to sediment addition. All others responded positively, probably due to habitat improvement afforded by the sediment in comparison to bare stones and also related to the stable flows during our experiment [21]. Motile and metaphytic algal forms increased their representation with added sediment, adding to evidence that these are strongly associated with fine sediment substrata [22]. Total algal cell density responded positively to added sediment, but not biomass measured as chlorophyll a, presumably because increases in cell densities primarily affected the smaller-bodied taxa comprising the motile and metaphytic growth forms [23] rather than their larger-bodied, chlorophyll-rich filamentous green counterparts.

Consistent with our prediction, but in contrast to most published literature (see review by Young et al. [24]), leaf decay increased with sediment addition, providing further evidence of this puzzling phenomenon (see Matthaei et al. [17]). While it is possible that sediment-related abrasion is at play, we witnessed no sediment movement during our experiment that involved moderate current velocities of less than 15 cm per second. Thus, we suspect this pattern is related to use of freshly collected leaf material that may still be alive [25]. Smothering by fine sediment may result in more rapid cell death and cuticle breakdown compared to non-buried leaves, with implications for processing of leaf material in streams receiving freshly fallen leaves (as in New Zealand or Australia, where the senescent leaves associated with deciduous trees are rare) and with high fine sediment loads [26].

As expected, nutrient enrichment generally acted as a subsidy, affecting about a third of all response variables in a positive manner and increasing total invertebrate abundance, algal cell density and biomass, but not organic decay rates. Invertebrate abundances of both pollution-tolerant (e.g. Chironomidae) and generally sensitive taxa (EPT) increased with nutrient enrichment, indicating that enriched levels were still within the range providing subsidy effects. Note that our nutrient concentrations correspond to moderate levels in New Zealand deer and dairy farming streams [10] but that higher anthropogenic levels occur elsewhere in the world [27]. Algal taxa that responded positively to enrichment tended to be those commonly associated with nutrient-rich conditions (species of *Nitzschia* and *Navicula*) [28] and taxa belonging to the metaphytic growth form (e.g. *Ankistrodesmus* and *Scenedesmus*), whose representation increased consistently with rising nutrients as filamentous forms decreased, indicating that metaphytic algae may proliferate more rapidly than filamentous algae with rising nutrients.

We also gained support for our prediction that the effects of raised water temperature would be generally positive and with community changes favouring taxa tolerant of elevated temperatures. Algae generally responded positively to raised water temperature with higher total cell density and biomass, and with changes in community composition favouring higher densities of the cyanobacterium *Cylindrospermum*, which is known to proliferate at higher temperatures [29]. By contrast, total EPT abundance decreased with warming, driven by lower abundances of *Deleatidium* and Hydrobiosidae, most likely due to thermal stress in these temperature-sensitive invertebrate taxa [30], while the abundance of the temperature-tolerant Nematoda [31] increased with warming. Leaf decay was not significantly affected by raised water temperature.

### Multiple Stressor Interactions

Nutrients and sediment interacted for 20% of variables (Table 3). The effects of nutrient enrichment changed from positive to negative at high sediment levels for the generally sensitive EPT taxa, including Conoescidae. This interaction may result from contrasting patterns of competitive dominance, with taxa such as chironomids, for example, performing consistently better at higher nutrient levels regardless of sediment conditions. Earlier studies in real streams [5] and a related streamside channel experiment [17] also reported that stronger negative effects of high sediment frequently overrode positive effects of nutrient enrichment on invertebrate variables. High nutrient and sediment levels interacted synergistically to yield higher cell densities of the diatom *Navicula cryptocephala*, which has the motile growth form generally considered to provide superior competitive ability in environments rich in nutrients or fine sediment [23]. The same pattern was seen for metaphytic algae, indicating that this growth form may also have a competitive advantage under high sediment and nutrient-rich conditions.

Raised temperature and nutrient enrichment interacted for 17% of variables (Table 3). This interaction was antagonistic for leaf strength loss, with raised temperature increasing strength loss at ambient but not enriched nutrient levels, in contrast to the synergistic effect observed by Ferreira & Chauvet [16]. Further, in all five invertebrate cases where nutrients and temperature interacted (EPT abundance, Chironomidae, *Oxyethira*, Conoescidae and Hydrobiosidae), abundances were highest at intermediate nutrient levels at ambient but not at raised temperature. Some invertebrates may gain an advantage from a moderate increase in nutrient concentration because of increased productivity of food resources (algae, fungi), but at higher levels of nutrient enrichment the advantage may be lost because non-diet taxa come to dominate. We can only speculate that when high nutrients and high temperature are combined, food productivity is increased without the loss of preferred algal species. In contrast to the invertebrate cases above, the alga *Ankistrodesmus* had its highest cell densities at intermediate nutrient levels at raised but not ambient temperature. These patterns show how biological responses to nutrient enrichment can follow a subsidy-stress gradient [13] but that such responses may also depend on temperature.

Interactions between temperature and sediment were the most common interaction type of all, affecting 34% of all response variables (Table 3). Raised temperature strengthened the negative effect of added sediment synergistically for both EPT abundance and *Gomphonema minutum* density; in channels without added sediment the effect of raised temperature was neutral for EPT abundance and positive for *G. minutum.* These findings support the notion of stress-induced sensitivity [32], whereby the negative effects of a primary stressor cause increased sensitivity to a second stressor. We would extend this notion to include a second factor that, on its own, may not have actually been acting as a stressor in the absence of the first factor. For filamentous algae, by contrast, a negative effect of sediment addition occurred at ambient but not at raised temperature (where sediment addition had little effect), indicating an antagonistic interaction. At ambient water temperature, sediment addition generally resulted in positive effects (for Chironomidae, algal cell density and *Encyonema minuta*) that frequently peaked at intermediate levels (total invertebrates, invertebrate taxon richness, Copepoda, *Oxyethira*, Tanypodinae, algal taxon richness and algal biomass). At raised temperature, however, added sediment had weaker positive (algal cell density, *Encyonema minuta*), neutral (Chironomidae) or, most often, negative effects (total invertebrates, invertebrate taxon richness, Copepoda, *Oxyethira*, Tanypodinae, algal taxon richness and algal biomass). Further, in channels without added sediment the effect of raised temperature was generally positive. It seems that fine sediment addition to an intermediate level acted mainly as a subsidy, providing additional habitat that increased algal and invertebrate abundance and richness, but only at ambient temperature. The putative subsidy was offset by a complex interaction with raised temperature for a wide range of population and community responses. These findings parallel the complex interactive effects of sediment addition and flow reduction reported by Matthaei et al. [17], where the effects of sediment worsened when combined with reduced flow, and provide further insight into the pervasive interactive effects of fine sediment with other stressors and the challenge these pose to management.

Our results show that population (individual taxa), community (taxon richness, etc.) and ecosystem variables (decay rate) can all be responsive to the focal stressors. When trophic levels are compared, algal variables were more frequently influenced than invertebrate variables by each of the three stressors (Table 3). We can imagine a relatively straightforward reaction of algae to increased nutrients (critical resource for photosynthesis) and temperature (metabolic rate control) while sediment may exert its pervasive effect mainly by providing favoured habitat. On the other hand, it is possible that added sediment may serve to reduce invertebrate grazing pressure, and the responses of individual algal taxa can also be expected to react to shifts in competitive status at different stressor levels. It is interesting to note that, based on significant factor main effects, invertebrate responses were less common than algal responses, whereas more invertebrate variables showed significant interactions among stressors than did algae. For invertebrate consumers, the underlying mechanisms of action of the studied stressors may be generally less straightforward than for algal producers. In many cases, invertebrate responses must be a complex response to the density patterns of algal taxa that are part of their diet, coupled with the effects of improving or degrading habitat conditions (fine sediment), physiological responses to temperature, and a shifting competitive hierarchy. Of particular interest is the similarity in patterns for algal and invertebrate taxon richness, which must at least partly reflect the basic importance of algae as a food resource for many of the invertebrates.

### Management Implications

Our streamside channel experiment has the advantages of combining rigorous control of focal stressors with sufficient power to detect effects and natural immigration and emigration rates of stream organisms, but it lacks realism in terms of its restricted spatial and temporal scales. Further, our study design had relatively few replicates at the level of the studied two-way interactions and did not permit investigating three-way interactions, and our experiment was run only once. Thus, extrapolation of our results to field conditions needs to be done with some caution.

Our key findings are that deposited fine sediment was the most pervasive of the studied three stressors and that a rise in water temperature of just 1.4°C, applied only on the 18 sunny days during our 30-day experiment, frequently interacted with augmented sediment and nutrients in complex ways. While temperature appeared less often as a main effect than the other stressors in our analysis, pairwise interactions of temperature with the other stressors were common. Indeed, temperature influenced 59% of all biological response variables as a main effect or in a significant interaction (in comparison to 59% for nutrients and 93% for sediment). Our heating treatment, which simulated temperature changes due to a loss of riparian shading from tussock grasses in an agricultural landscape, caused algal proliferation and declines in the abundances of EPT invertebrate species that are used worldwide as sensitive indicators of ecological stream health. Areas where agricultural development involves removal of forest rather than tussock might experience even stronger effects because forest removal would lead to greater temperature increases.

The fact that taxon richness of both invertebrates and algae responded to the interactive effects of water temperature and fine sediment highlights the potential importance of this particular interaction for biodiversity and multiple levels of community organization. In addition, the decay rate of leaf material, increasingly used as an indicator of stream health [24], accelerated with nutrient enrichment at ambient but not at raised temperature. Thus, managers should pay particular attention to the removal of riparian shading from streams already subjected to high fine sediment inputs, or to land-use changes that increase erosion or nutrient runoff in a landscape without riparian buffers, because these may have particularly unfortunate consequences for stream health. We emphasize the likely importance of intact or restored buffer strips, both in reducing fine sediment input (the most pervasive stressor of all) and in maintaining cooler water temperatures.

Our studies of individual and combined effects of multiple stressors in grassland streams have used surveys and manipulative experiments covering a range of scales and stressors (e.g. [5], [17]). Based on our combined findings, we can definitely conclude that consequences of multiple stressors are often unpredictable on the basis of knowledge of single effects. Consequently, for the integrated management of streams and their catchments to be meaningful, management decisions need to be informed by knowledge of the interactive effects of the particular multiple stressors that are operating

## Materials and Methods

### Ethics Statement

All necessary permits were obtained for the described research. This included resource consents 2005.725 and 2006.070 from the Otago Regional Council, Maori consultation with Te Runanga o Moeraki, land and river access permission from the Department of Conservation and private landowners Jan and Clyde Douglas. All biological material was collected under a permit issued by the Ministry of Agriculture and Fisheries to the University of Otago, Department of Zoology.

### Study Site

The study was conducted during Austral summer from 11 January to 3 March 2007 in experimental streamside channels installed on the floodplain of the Kauru River, a third-order stream in the Otago province of New Zealand (170°44.6′ East, 45°6.5′ South, 98 m a.s.l). Land use is mainly sheep and beef cattle farming at low stock densities. The river water is relatively nutrient-poor (see non-enriched nutrient values below) but contains diverse and abundant algal [33] and invertebrate communities [34].

### Experimental Design

**Figure 6:**
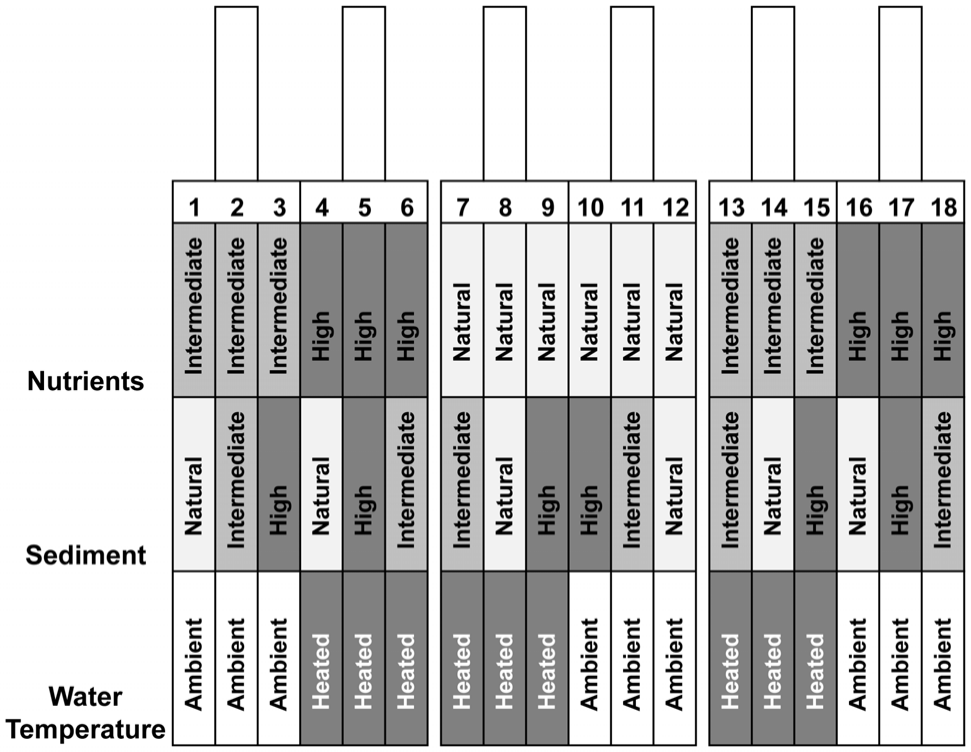
Diagram of the experimental design, showing the 6 inflow pipes (above) and the combinations of ambient and augmented stressors applied to each of the 18 channels.

We manipulated nutrients, fine sediment cover on the bed and water temperature in 18 channels using 3 nutrient levels × 3 sediment levels × 2 temperature treatments in a factorial design (Fig. 6). Six sets of three channels made of steel sheet (250×15×15 cm) were installed on a flat gravel area between two river braids. River water was supplied through PVC pipes (Humes Pipeline Systems, Dunedin; diameter 15 cm) and inflow and outflow weirs equalised flow and water depth across channels. Mean discharge and mean current velocity per channel, measured on 6 dates, were 1.1±0.2 L s^−1^ and 12.3±0.3 cm s^−1^ (standard errors; n = 108), and mean water depth was 7.0±0.1 cm (measured on 7 dates; n = 126). This velocity is towards the lower end of the range of local current speeds found in the Kauru River at baseflow [34]. Three of the six supply pipes were connected to gas-fired water heaters (model 16H; Bosch, Stuttgart, Germany) and pumps (model JR.2; LINZ Electric, Verona, Italy) that added heated water to the flow entering ‘heated’ channels. To prevent clogging, a 50 mm mesh fence was erected upstream of the supply pipe intakes and cleaned every 3 days.

The experimental channels formed an open system with the river allowing natural immigration and emigration of organisms. Each channel contained 3 cm of mixed gravels (16–64 mm width) from a nearby dry riverside channel. Flow began on 11 January and the channels were colonised by drifting algae and invertebrates for 21 days. On 31 January, each channel received one standard load of invertebrates, to augment natural colonisation of taxa underrepresented in the drift. This standard load was obtained from the adjacent river by kick-net sampling for 3 minutes (mesh size 200 µm) from a 0.36 m^2^ bed patch (comparable to the 0.38 m^2^ channel area). Samples were collected moving upstream in a uniform area of riverbed and assigned randomly.

On 1 February (day 0), the three nutrient treatments were assigned randomly to the six supply pipes, each feeding three channels (Fig. 6). Nutrients (NaNO_3_ plus KH_2_PO_4_) were supplied continuously for 30 days using battery-driven fluid-metering pumps (model QBG; Fluid Metering Inc., Syosset, New York) to achieve concentrations found in deer farming streams (intermediate level; six channels, means achieved, measured on days 0, 5, 10, 15, 20, 25 and 30, of 67.7±1 µg L^−1^ for nitrate-N and 41±0.8 µg L^−1^ for phosphate-P; n = 84) or dairy farming streams (high level; six channels, means 379±2.1 µg N L^−1^ and 222.9±2.5 µg P L^−1^) in Otago [5]. Six channels were not enriched (natural level; means 2.4±0.4 µg N L^−1^ and 1.9±0.1 µg P L^−1^).

The raised temperature treatment was applied to half the channels (selected within each of three spatial blocks consisting of six channels each to complement the randomly assigned nutrient treatments; Fig. 6) from 10.00 h to 19.00 h on predominantly sunny days (to approximate a natural pattern of water temperature increase in streams lacking upstream riparian cover). On the 18 days when heated water was applied, ambient temperature was raised in the heated channels by a mean of 1.4±0.2°C for 8.9±0.02 h per day (n = 18), with a mean daily maximum temperature of 23.6±0.2°C in heated and 22.2±0.2°C in control channels (n = 162). This increase is approximately equivalent to a decrease in stream shade of 28% (see Fig. 7 in Rutherford *et al.* [35]). We also measured shade in two second-order Otago grassland streams with contrasting riparian tussock cover as percentage of diffuse non-interceptance received at the water surface as a proportion of the incident light of a uniform sky [36]. This was accomplished on an overcast day by taking matched instantaneous measurements with photosynthetically available radiation sensors (LI-190, LI-COR Inc, Lincoln, USA) at 5 m intervals along a 50 m reach (at 25, 50 and 75% of the stream width) at the water surface and in an open reference site two meters above the stream bank and riparian vegetation. The stream in ungrazed tussock was 84% shaded while the one in intensively grazed tussock with no tussock margin was 52% shaded, a reduction of 32% and close to that predicted by Rutherford *et al*. [35] to result in a 1.4°C increase in maximum water temperature.

Sediment treatments were assigned within nutrients and temperature treatments, in a manner that complemented the nutrient by temperature combination in each channel to result in the 18 different combinations of all three factors required to create a balanced study design (Fig. 6). Fine river sand (grain size 0.2 mm; [10]) was added on day 0, resulting in sediment values corresponding to those occurring in dairy (intermediate; six channels, means achieved measured on days 5, 10, 15, 20, 25 and 30 of 81.2±1.5% bed cover and 4.9±0.3 mm sediment depth; n = 108) or deer farming streams in Otago (high; six channels, means 93.9±1.3% cover and 18.5±0.7 mm depth) [5], [10]. Six channels had no sediment added (natural; means 2.5±0.4% cover, 0.2±0.02 mm depth).

Nutrient, temperature and sediment measures differed as required among the corresponding experimental treatments (nested repeated-measures ANOVAs: *P*≤0.001, Tukey HSD tests: *P*≤0.001).

### Biological Response Variables

On day −1, we introduced six terracotta tiles (10×10×1.4 cm; as standardized substrata for invertebrates) and six leaf packs per channel, two each in the upstream, middle and downstream thirds. We used 10 g (fresh mass; 10–15 leaves bolted together) of the evergreen native riparian shrub mahoe *Melicytus ramiflorus* Forst & Forst [17], held in place by surface stones.

On days 18 and 30, one tile and one leaf pack were sampled from each channel third by lifting quickly into a hand net (frame 20×15 cm; mesh size 250 µm) and then transferring contents to sealable plastic bags. Where tiles were beneath the sediment, the associated sediment was also taken. On day 29, benthic algae were sampled within each channel third using a plastic ring (3 cm diameter) placed at random. Algae were either scraped with a razor (on stones) or sucked up with a pipette (on fine surface sediment) from within the ring. Samples were topped up to 50 ml with stream water and divided in half for analysis of biomass and community composition, the latter being fixed in 4% formalin. All samples were placed on ice in the dark, and non-preserved samples were frozen in the laboratory the same day and stored at −18°C until processing.

### Laboratory Work

Invertebrates were gently washed from tiles after thawing and retrieved in a sieve (mesh size 200 µm), then stored in 70% ethanol prior to identification to the lowest practical taxonomic level using a dissecting microscope (Olympus SZ51, 8–40×, Tokyo, Japan) and the standard taxonomic key for New Zealand’s aquatic invertebrates [37].

Leaf packs were defrosted and rinsed. Remaining leaf biomass was determined as ash-free dry mass using standard methods [38] and expressed as a percentage of the average of ten leaf packs treated identically immediately after leaf collection (day −3). Leaf strength was determined as the mass required to force a blunt metal pin through the leaf [24]. One measure was made per leaf (randomly but avoiding veins) for five randomly selected leaves per pack, and leaf strength was expressed as percentage of the average strength of 25 fresh leaves determined on day −3. Due to almost complete decay of leaf packs by day 30, only day 18 results are presented.

Algal biomass as chlorophyll a was determined spectrophotometrically using standard methods [28], [38]. Algal community samples were sonicated (Elma Transonic 10/40H, Singen, Germany) at 40 Hz for 2 min in an ice water bath to detach cells from sediment particles and then homogenised with a blender (Omni Mixer, Ivan Sorval Inc., Newton, USA). For each cell count, approximately 300 cells (mean achieved 291±8) were identified at 400× magnification to the lowest practical taxonomic level using an inverted microscope (Zeiss Axiovert 25, Jena, Germany). Where algal filaments were present and cells were difficult to discern, 10 µm increments were counted. Algae were also classified according to growth form after Schneck et al. [20]: (i) adnate or prostate, (ii) erect or with mucilaginous stalks, (iii) motile, (iv) filamentous and (v) metaphyton (Table 4).

**Table 4:** Algal growth form classification.

**(i) ADNATE OR PROSTATE**
Species of *Achnanthidium, Cocconeis, Epithema, Planothidium, Rhoicosphenia, Rhopalodia, Rossithidium, Gloeocystis, Chamaeosiphon.*
**(ii) ERECT OR WITH MUCILAGINOUS STALKS**
Species of *Cymbella*, *Encyonema, Gomphoneis, Gomphonema* (excluding *G. parvulum* and *G. minutum,* which we classified as adnate as suggested by (Passy 2007), *Synedra* and *Fragilaria*).
**(iii) MOTILE**
Species of *Frustulia, Navicula, Nitzschia, Pinnularia, Surirella* and *Stauroneis*).
**(iv) FILAMENTOUS**
Species of *Diatoma, Melosira, Oedogonum, Spirogyra, Stigeoclonium, Geminella, Rhizoclonium, Ulothrix, Anabaena, Cylindrospermum, Oscillatoria, Phormidium, Coelodesmium* and a non-identified filamentous cyanobacterium).
**(v) METAPHYTON (ALGAE WITHOUT A FIXATION STRUCTURE)**
Species of *Staurastrum, Cosmarium, Closterium, Ankistrodesmus, Chlorella, Scenedesmus, Selenastrum, Pediastrum, Trachelomonas* and *Chlamydomonas*).

Classification of algae according to growth form into five groups after Schneck et al. [20].

### Data Analysis

All analyses (nested ANOVAs) were conducted in PASW Statistics 18.0 (SPSS: An IBM Company, Chicago, USA). Data were log-transformed where necessary to improve normality and homoscedasticity. Nutrients, sediment and temperature were fixed main factors. Sample (positions 1–3, in each channel third) was a fixed (as opposed to a random) nested factor [39]. This was done because, based on a previous experiment in the same channels [17], we expected the physical habitat for stream organisms (e.g. cover and thickness of deposited fine sediment) to change to some extent with distance down the channel as current velocity tended to be somewhat faster at the inflows.

For algal and leaf pack data (single sampling date), the ANOVA model was intercept (d.f. 1) + nutrients (2) + sediment (2) + temperature (1) + nutrients×sediment (4) + nutrients×temperature (2) + sediment×temperature (2) + sample (nutrient) (6) + sample (sediment) (4) + sample (temperature) (2) + error (28, n = 54; see Matthaei et al. [17] for a similar model). For the invertebrate data (collected on days 18 and 30), we used the repeated-measures equivalent of this model (with sampling day as the within-subjects factor; see Herrmann et al. [40] for a similar model). One invertebrate sample on day 30 was lost.

We selected the Type I (sequential) sums of squares, the appropriate method for analysing this type of nested design in PASW/SPSS [41], [42]. Note that our model lacks a term testing three-way between-subjects interactions because with just one channel replicate of each three-factor treatment combination this interaction term could not be separated from the residual error [39]. This limitation implies that our analysis is likely to somewhat underestimate the actual frequency of significant single-factor effects and two-way interactions [42].

To assess effects on invertebrate and algal communities we performed nested MANOVAs (with the multivariate equivalents of the models above) on densities of taxa that were present in at least 50% of all samples and, for algae, also contributed at least 1% of the total community. We subsequently examined the between-subjects effects for each individual taxon and the within-subjects effects for all common invertebrate taxa. Algal growth form compositions were analysed using a similar MANOVA with subsequent examination of between-subjects effects for each growth form. Box’s Tests of Equality of Covariance Matrices confirmed that the data fulfilled the assumptions of these MANOVAs.

If between-subjects effects were significant, pairwise comparisons were performed for the factors nutrients and sediment using post hoc tests (Tukey’s HSD). Where significant interactions are present, interpretation of the main effects of the experimental factors concerned must be done with care. Therefore, we followed Quinn and Keough’s [39] recommendation that individual main effects should be interpreted only where the effect size of the interaction is smaller than the size of the corresponding main effects, which was the case for the majority of response variables. All exceptions from this rule are identified in the Results tables. When examining within-subjects effects on invertebrate response variables, results were corrected using the Greenhouse-Geisser method [39] in cases all cases where the assumption of sphericity (examined with Mauchly’s tests) was violated. For the sake of brevity, the results for the nested factor sample are not presented (see Matthaei et al. [17]). Including this factor enabled us to quantify within-channel variation and improve overall predictive power, but significant sample effects merely indicate that responses differed between channel thirds across all channels. This is irrelevant to our research objectives. Similarly, the results for the invertebrate repeated-measures factor time are not presented because we were not interested in changes across time per se.

Significance level for all tests was P<0.05. Adjusting the significance level to control the family-wise Type I error rate in sets of related statistical tests is regarded increasingly as too conservative by both ecologists and statisticians [39], [43], [44]. Therefore we present standardised effect sizes (partial eta^2^ values, range 0–1; [42]) for all findings with P≤0.05 instead, as recommended by Nakagawa [44], to allow readers to evaluate the biological importance of each individual result.
